# Doctors and the Etiquette of Mobile Device Use in Trauma and Orthopedics

**DOI:** 10.2196/mhealth.4122

**Published:** 2015-06-26

**Authors:** Oliver Blocker, Lydia Hayden, Alison Bullock

**Affiliations:** ^1^ The Cardiff Unit for Research and Evaluation in Medical and Dental Education (CUREMeDE) Cardiff University Cardiff United Kingdom

**Keywords:** education, medical, cell phones, patient-physician relationship

## Abstract

**Background:**

The etiquette surrounding the use of mobile devices, so-called "mobiquette," has been previously identified as a barrier to use in an educational context.

**Objective:**

To investigate the influence of mobile device use on patient and staff opinions in the trauma and orthopedics department at a teaching hospital in Wales.

**Methods:**

A survey of patients at the bedside and staff in their work environment was undertaken. Data included age, frequency of observed use, suspected main reason for use, and whether doctors’ use of a mobile device positively or negatively influenced participants' opinions of them as a professional and as a person.

**Results:**

A total of 59 patients and 35 staff responded. The modal age range was 40 to 54 years old. Most patients (78%) never see doctors using mobile devices in the workplace, compared with 3% of staff. The main reason for use was thought to be "communicating with colleagues" (48%) followed by "Internet use/applications for work reasons" (40%). Approximately 40% of patients' opinions of doctors were positively influenced by device use, compared with 82% of staff. This difference between patient and staff opinions was statistically significant for both professional (*P*<.001) and personal (*P*=.002) opinions.

**Conclusions:**

Patients are likely to have a negative opinion of doctors using mobile devices in the workplace. This can be balanced by the more positive opinions of colleagues. We advise doctors to remember "mobiquette" around patients.

## Introduction

Mobile technology is being used with the intention of enhancing the learning of medical students and doctors in the workplace and the evidence of its value is growing [1,2]. What remains under-researched is the opinions of patients and colleagues regarding doctors’ use of mobile devices for learning on the ward. The term “mobiquette” was coined by Ellaway and Masters in 2008 [3] to describe the etiquette of mobile device use and appropriate mobiquette has been identified as a challenge to device use in the workplace [1,2]. Even if there was consensus about what is considered “appropriate,” there is concern that learners’ (eg, students and trainees) interactions with devices will be misinterpreted. Without looking over a user’s shoulder, we cannot know if the mobile technology is being used for professional, educational purposes or personal reasons (ie, texting, social media, or Internet browsing). Beyond the etiquette issue, another concern is the fear of superficial learning [4] and the erosion of the traditional practice of internalizing knowledge, replacing it with an ability to locate information in “the cloud.” If the concept of “I may not know the answer, but I know where to find it” becomes more prevalent in medicine, it may require a change in both doctors’ and patients’ perceptions of practice. Given these negative associations, it would be reasonable to have measures in place to ensure that mobiquette is observed by those using mobile technology in a clinical environment. The iDoc project [1], for example, advises its participants to inform their colleagues that they will be using mobile devices to retrieve clinical information and to reassure them that their use is work related. When using a mobile device in front of patients, trainees are advised to inform them what they are doing and to potentially involve patients in the process by sharing a view of the information on the screen. However, importantly, users need to exercise judgement around patients [5]. Professionalism, which includes establishing and maintaining partnerships with patients and colleagues [6], extends to the various situations faced by a doctor, including judgements about whether and how to share information from a mobile device.

Too readily assumptions are made about mobile technology, its users, and people’s opinions about device use. Doctors now in training are mainly in their 20s and early 30s and can be included in the demographic group known as “millennials” [7] or the “net generation” [5], but it should not be assumed that they have the technical skills or attitudes to use mobile technology appropriately around other people. Age may also be unrelated to the opinions people form about mobile device use. Part of the purpose of the research we report here was to explore these assumptions by examining the relationship between patient and staff ages and their opinions of mobile device use.

Our primary research question was, “Does mobile device use influence patient or staff opinions of doctors?” Our null hypothesis was that across our respondents as a whole, mobile device use would not influence group opinions either positively or negatively and that there would be no difference between patients’ and staff members’ views. Our secondary research question was, “Is there a relationship between what patients and staff believe devices are being used for and age?”

## Methods

### Overview

We used a survey of patients at the bedside and staff in their work environment. A hard copy questionnaire (Multimedia Appendix 1) was issued face-to-face by OB (who surveyed staff) and LH (who surveyed patients) on 1 day in September 2013. The respondents completed the questionnaire in front of the doctor-researcher and were given an opportunity to clarify any questions.

### Setting and Sample

The setting was a trauma and orthopedics department at a teaching hospital in Wales where the authors (OB and LH) were doctors in training. Participants were a convenience sample of inpatients on 2 adult orthopedic wards and the staff working in various environments in the orthopedics department of the same hospital. These environments included the operating theater, fracture and elective outpatient clinics, inpatient wards, and secretarial and management departments. There were practical reasons for the discrepancy in the environments for the patient and staff populations: patients on the ward are easily sampled in reasonably large numbers and usually have time to talk to doctor-researchers. In the theater environment the majority of patients are anaesthetized and undergoing an operation, and it was judged inappropriate to survey patients in the orthopedics clinic. There was a high patient-to-staff ratio on the ward and ward staff comprised 2 main groups (nursing and therapy staff). Therefore, to ensure adequate staff numbers on the study date and to expand the variation of staff groups, the surveyed staff population was extended to junior and senior grades of all health care professionals in the multidisciplinary team found throughout the orthopedics surgery department. The exception to this was doctors who had completed their training, as the survey was related to opinions of doctors currently in training.

### The Survey Instrument

Data on age, frequency of observed use, and main reason for device use were collected. Age was presented in 15-year ranges from “less than 25 years” to an upper age range of “85 plus years.”

Frequency of observed use was classified as regularly, occasionally, and never. Suspected main reason for device use was a single tick box from a selection of 6 options (see Textbox 1).

**Suspected main reason for mobile device use given as options in the questionnaire.**

Suspected main reason for device use:

1. Communicating with friends
2. Social media/Facebook
3. Gaming
4. Internet use for personal reasons
5. Communicating with colleagues
6. Internet/electronic textbooks/medical apps for work reasons

The first 4 options cover common uses for connected, mobile devices for nonwork reasons (the hospital in this survey had open-access Wi-Fi for both patients and staff). We recognize that options 1-3 are a subset of option 4 (“Internet use for personal reasons”). We purposefully sequenced these items so that option 4 would pick up other forms of personal Internet use, such as shopping. Device use for professional education includes using electronic textbooks stored on a device, mobile applications (eg, a medical calculator), or accessing Internet-based medical information (eg, UpToDate). Communication with colleagues using a connected mobile device (ie, one that’s connected to the Internet via Wi-Fi or a mobile telephone network) could include use of messaging systems.

### Opinion Questions

To determine a respondent’s opinion regarding a doctor using a mobile device in their presence, 2 questions were asked:

1. How does a doctor using a phone at work/the bedside influence your opinion of them as a professional?
2. How does a doctor using a phone at work/the bedside affect your personal opinion of them?

These were closed questions with the response options of “positively” or “negatively.” The 2 questions therefore addressed potential differences in interactions—between the professional (views on the trainee as a doctor) and the personal (views on the trainee as a person). A “don’t know” option was not included. While we appreciated that this may have forced respondents to express an opinion that they did not hold, we assumed that the sample population (ie, patients over the age of 16 and staff members over the age of 18) would certainly have encountered mobile devices and would have some opinion about their use around other people. The literature on research methodology would suggest that data quality is not enhanced by the inclusion of a “don’t know” option [8].

### Analysis

All data were analyzed in SPSS (IBM SPSS Statistics for Macintosh, Version 20.0). Statistical tests of significance (Chi-square and Fisher’s exact) were used to test difference between patient and staff responses. Correlation was tested using Pearson’s 2-tailed test of significance.

### Ethical Considerations

Using the National Health Service’s (NHS) Heath Research Authority online decision tool [9], we determined that this project was not classified as research so approval to conduct the survey was sought from and granted by the trauma and orthopedics department. The data were collected from a volunteer sample. All participants were assured of confidentiality and anonymity. Verbal consent to participate was obtained. Privacy was enhanced by using a paper questionnaire on a clipboard that could be closed over, concealing their responses from others in the room.

## Results

There were 94 respondents in total; 59 patients and 35 staff members. Five patients and 9 staff members required explanation of the opinion questions.

The modal age range for all respondents was 40 to 54 years old. For patients, the modal range was 70 to 84 years old (27% of patient respondents). There were 4 patients over age 85, 2 who were 90 years old, and 2 who were 91 years old. The modal range for staff was 40 to 54 years old (60% of staff respondents). There were no staff members over 69 years old.

The results for the frequency of observed use by patients at the bedside and staff in the work environment are shown in Table 1.

**Table 1:** Results for frequency of observed use of devices in the workplace/bedside by patients and staff.

Frequency of observed use	Patient % (n)	Staff % (n)	Total % (n)
Regularly	2% (1)	46% (16)	20% (17)
Occasionally	20% (10)	51% (18)	32% (28)
Never	78% (40)	3% (1)	47% (41)
Missing data	8	0	8

The results for observed frequency of use were notably different for the 2 groups: 78% of patients compared with 3% of staff never saw doctors use mobile devices, and 2% of patients compared to 46% of staff regularly saw doctors using mobile devices in the workplace. These results were significantly different (Chi-square, *P*<.001).

For all respondents, the top suspected main reasons for use was thought to be “communicating with colleagues” (48%) followed by “Internet use/applications for work reasons” (40%) (Table 2). There were no significant differences between patient and staff groups for the suspected main reason for use (Chi-square, *P*=.335). Neither patients nor staff suspected that doctors were using social media or games on their devices.

**Table 2:** Patients’ and staff members’ suspected main reason for mobile device use in the workplace.

Perceived main reason for use	Patients% (n)	Staff% (n)	All respondents% (n)
Communicating with colleagues	47% (27)	52% (17)	48% (44)
Internet/electronic textbooks/ medical apps for work reasons	43% (25)	33% (11)	40% (36)
Internet use for personal reasons	9% (5)	6% (2)	8% (7)
Communicating with friends	2% (1)	9% (3)	4% (4)
Social media/Facebook	0	0	0
Gaming	0	0	0
Missing data	1	2	3

Overall, 42% of patients’ opinions of doctors as a professional were positively influenced by device use, compared with 82% of staff (Table 3). Of total respondents, their professional (57%, n=53) and personal (56%, n=52) opinions of doctors were overall positively influenced by mobile device use. There was strong correlation between the results for the 2 opinion questions (Pearson’s correlation 2-tailed, *P*<.001); only 1 respondent gave differing answers.

**Table 3:** Influence of opinion of doctor results, by group.

Group	Influence opinion of doctor as a professional		Influence opinion of doctor as a person		Total No. (missing data)
	Positively	Negatively	Positively	Negatively	
Patient	42% (25)	57% (34)	44% (26)	56% (33)	59 (0)
Staff	82% (28)	18% (6)	76% (26)	24% (8)	34 (1)
Totals	57% (53)	43% (40)	56% (52)	44% (41)	93 (1)

The opinions of staff and patients differed greatly, with significantly more patients than staff being negatively influenced by mobile device use. This was true for both patient and staff opinions of doctors as professionals (57% vs 18%, Fisher’s exact test 1-sided, *P*<.001) and their personal opinions of doctors (56% vs 24%, Fisher’s exact test 1-sided, *P*=.002). Age and opinions were investigated, but due to the difference in the modal age ranges for patients and staff, these ranges were collapsed into 2 groups; under 55 years old and 55 years old and older. The patients’ split between these 2 groups was relatively balanced (44%, n=26, in the under age 55 group). In contrast the majority of staff were in this group (89%, n=31). No significant differences were found in the combined patient and staff group for professional (Fisher’s exact test 1-sided, *P*=.063) nor personal opinions (Fisher’s Exact test 1-sided, *P*=.087). We repeated the analysis for the patient-only group, and no significant relationships were found (Fisher’s exact test 1-sided, for professional opinion *P*=.398; for personal opinion *P*=.291). We did not repeat this analysis for staff due to low numbers in the over age 55 group.

## Discussion

### Principal Findings

It would appear from this study that most patients in the orthopedics wards are not observing doctors in training using mobile devices at the bedside, whereas staff members on the wards and in other work environments are seeing them used regularly. This would fit with the concerns that doctors have about using mobile devices in front of patients [1] and also the workplace environment, which means that staff members, including doctors, share the same nonclinical work spaces where mobile devices are commonly used. When they do see mobile devices being used, the majority of both patients and staff believe that their use is for work-related communication (48%) or educational reasons (40%). It was encouraging that no respondents thought mobile devices were being used for gaming or social media, but it is important to remember that the uses were suspected or perceived by the respondents and not actual, observed use. Unless mobile device use is being directly observed in close proximity (such as looking over a user’s shoulder) it is almost impossible to tell if a Web browser is showing Facebook or an online textbook. This result would suggest that when both patients and staff members do see doctors using mobile devices, they assume that their use is work-related. The authors suspect that in real life, this may not always be the case.

While for approximately 40% of patients their opinion of the doctor was positively influenced by mobile device use, this result was half that of the staff group (82%). In this study, the majority of patients’ opinions were negatively influenced by device use. Compared to staff, patients were significantly more likely to have their opinions of doctors both as professionals and as people negatively influenced by mobile device use. The patient group was more heterogeneous than the staff group, who as health care professionals were more likely to be using mobile devices in the workplace for the same reasons as doctors. They may have greater insight into how mobile devices can support doctors’ work and, therefore, would be less likely to form negative opinions of their use.

The relationship between the influence of device use on the respondents’ opinions of doctors as professionals and as people would suggest that these opinions are similar. The negative influence of device use on patients’ opinions is countered by the more positive influence it has on the opinions of colleagues. The lack of significant relationships regarding age and opinion is noteworthy; it debunks an assumption that opinions regarding mobile device use are age related.

### Strengths

One of the strengths of this study is that it is rare for patients’ opinions to be sought on these matters. Much of the current research is focused on doctors’ use of mobile technology [1,2,10,11]. This is 1 of few, if any, known studies to investigate patients’ (and other health care professionals’) opinions. The study is suitably powered; it is generally accepted that Fisher’s exact test requires samples of 30 in the groups being compared. A retrospective power calculation was performed on the approximate combined differences between patient and staff responses for the opinion questions, using the formula devised by Lehr [12]. If the power of the proposed hypothesis test is fixed at 80% and the level of significance of the 2-tailed test set at 5%, the number needed in each group is 25.

### Limitations

The limits of this study are the narrow population group; 1 department in 1 hospital in Wales and a convenience sample of both groups. It would be inappropriate to extrapolate the results to all patients and all doctors in the NHS in the United Kingdom. There is also an element of researcher bias, as a result of the face-to-face distribution of the survey. The researcher (OB) was known to all staff participating in the study. Bias was minimized by emphasizing that there were no right or wrong answers and ensuring confidentiality and anonymity. Five patients and 9 staff members required explanation of the opinion questions. The most common comment on the survey instrument was about the lack of a “don’t know/no opinion” option. While there were no refusals to participate among patients, not all staff members in each environment visited on the study day were available to participate, and 1 staff member refused to answer the opinion questions.

### Conclusions

Observing doctors using mobile devices was viewed negatively by a majority of patients, but positively by most staff members. Yet the majority of respondents thought that the main reasons for mobile device use was for work-related information retrieval and communication. No respondent thought doctors were using devices inappropriately for gaming or social media. Given the perception of appropriate mobile device use, it is interesting that this did not positively influence the opinions of patients. These opinions about device use showed no relationship to the age of the respondent. This is important. It reinforces the danger of making age-specific assumptions.

Doctors are using mobile devices at work but not in front of patients. Does the discrepancy between perceived appropriate use of devices and negative influence on patient opinions mean that patients need a better understanding of mobile technology in the workplace? Can doctors play a role in this? We recommend that doctors continue to be advised to be mindful of the etiquette regarding mobile device use in front of patients and colleagues.

## Appendix

Multimedia Appendix 1 Survey instrument.

Multimedia Appendix 2 Presentation from Medicine 2.0 2014, Malaga.
