# Supplementary material for: Doctors and the Etiquette of Mobile Device Use in Trauma and Orthopedics
Source: JMIR Mhealth Uhealth. 2015 Jun 26;3(2):e71. doi: 10.2196/mhealth.4122 (PMC4526965; doi:10.2196/mhealth.4122)
Supplement: Multimedia Appendix 1 [file mhealth_v3i2e71_app1.pdf]

The etiquette of mobile device use by doctors in Trauma and Orthopaedics

Thank you for helping us with our research into mobile device use in the workplace:

1. Are you?

A patient *A staff member*

2. What is your age? Please circle:

Less than 25 *25-39* *40-54* *55-69* *70-84* *more than 85*

4. How often have you seen a doctor using their phone/mobile device in your work environment? (Circle one response)

Regularly *Occasionally* *Never*

5. What do you think is the MAIN reason for a doctor using their phone/mobile device at work? (Tick ONE box only, please)

Communicating with friends	*Social media/Facebook*	*Gaming*
Internet use for personal reasons	*Communicating with colleagues*	*Internet/Electronic textbooks/medical apps for work reasons*

6. How does a doctor using a phone at work/ the bedside influence your opinion of them as a professional?

Positively *Negatively*

Comments:

5. How does a doctor using a phone at work/ the bedside influence your personal opinion of them?

Positively *Negatively*

Comments:

Thank you!

oliver.blocker@wales.nhs.uk
