# Supplementary material for: Doctors and the Etiquette of Mobile Device Use in Trauma and Orthopedics
Source: JMIR Mhealth Uhealth. 2015 Jun 26;3(2):e71. doi: 10.2196/mhealth.4122 (PMC4526965; doi:10.2196/mhealth.4122)
Supplement: Multimedia Appendix 2 [file mhealth_v3i2e71_app2.pptx]

## Slide 1
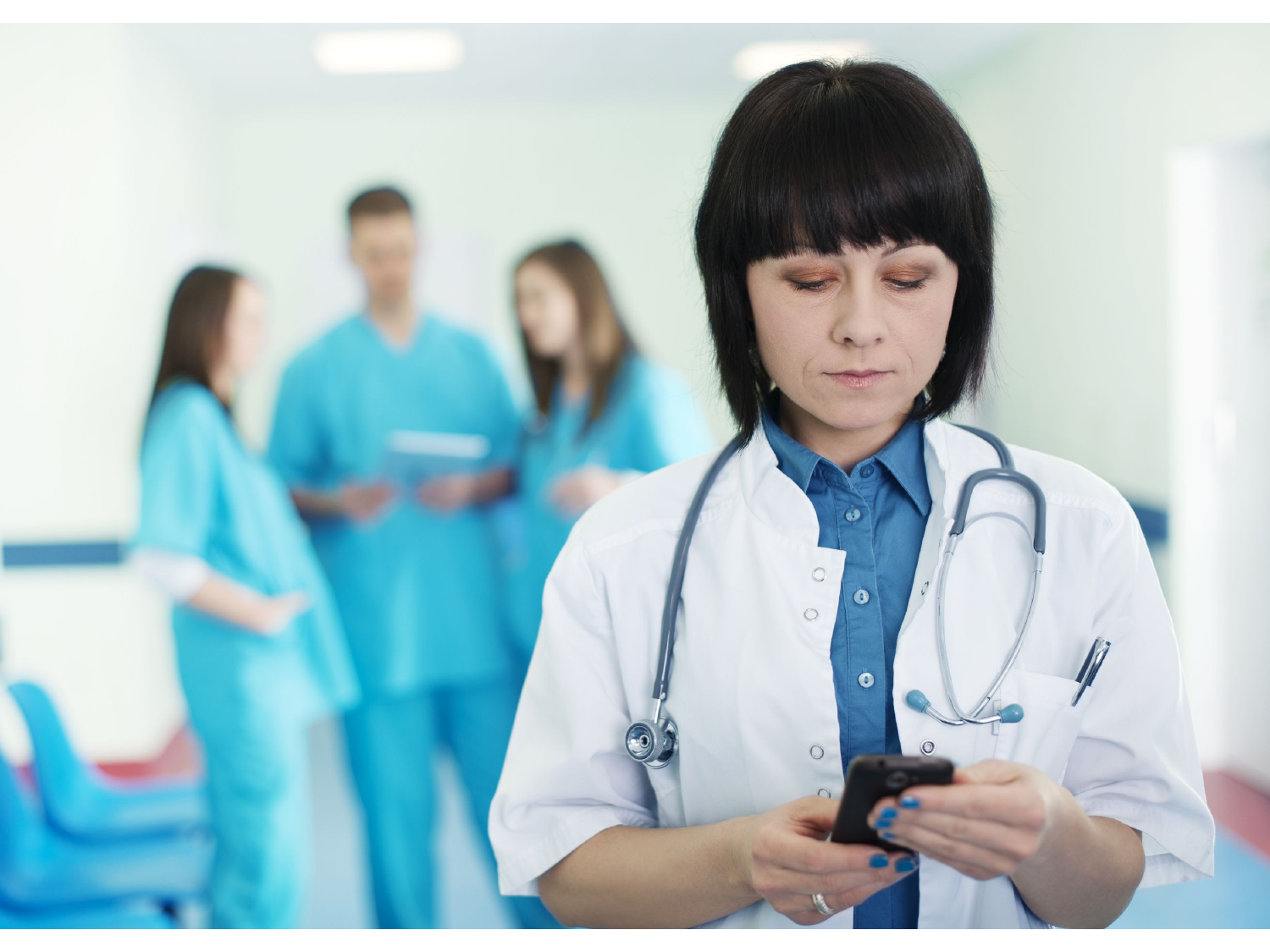

## Slide 2
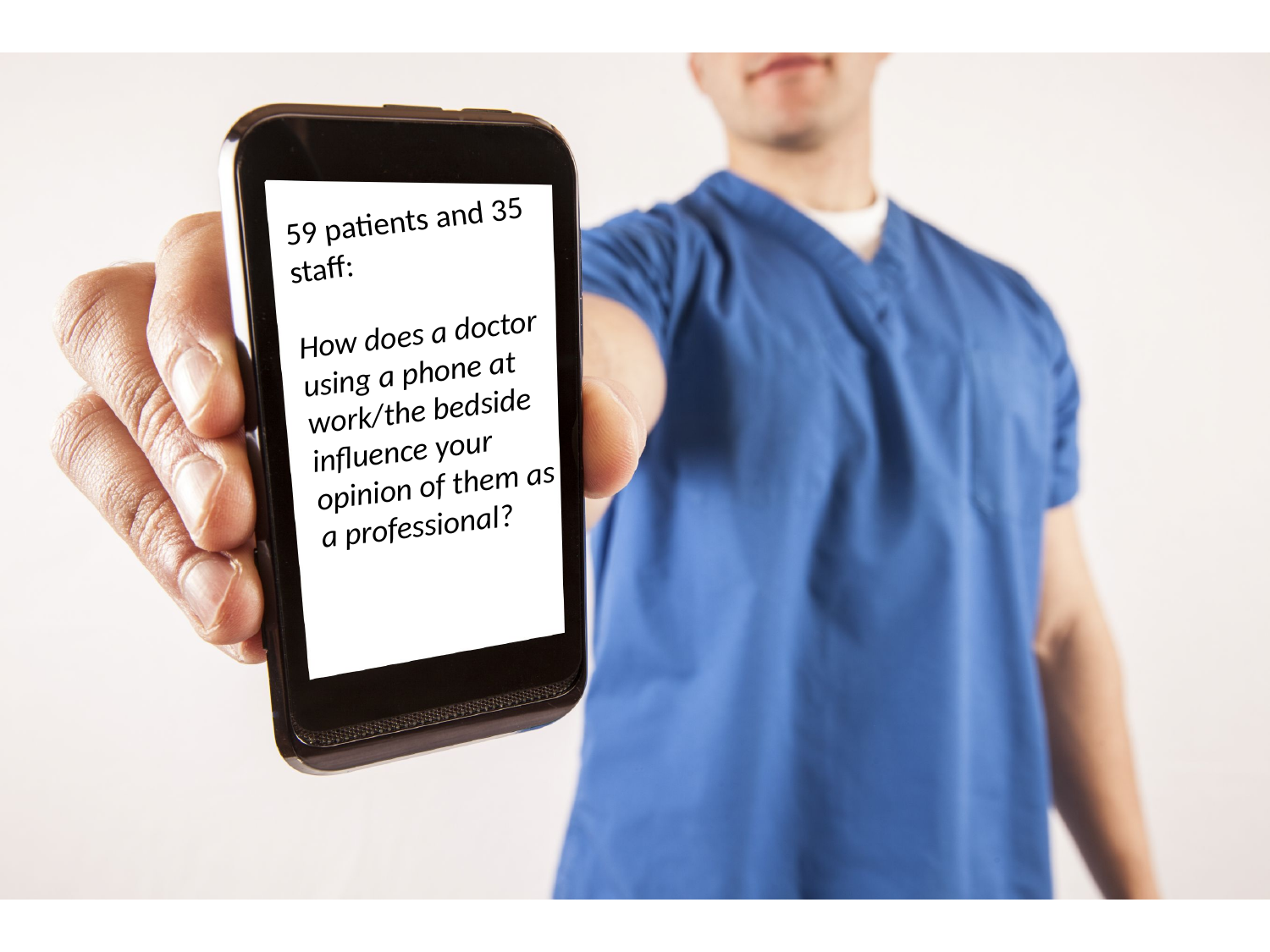

59 patients and 35 staff:
How does a doctor using a phone at work/the bedside influence your opinion of them as a professional?

## Slide 3
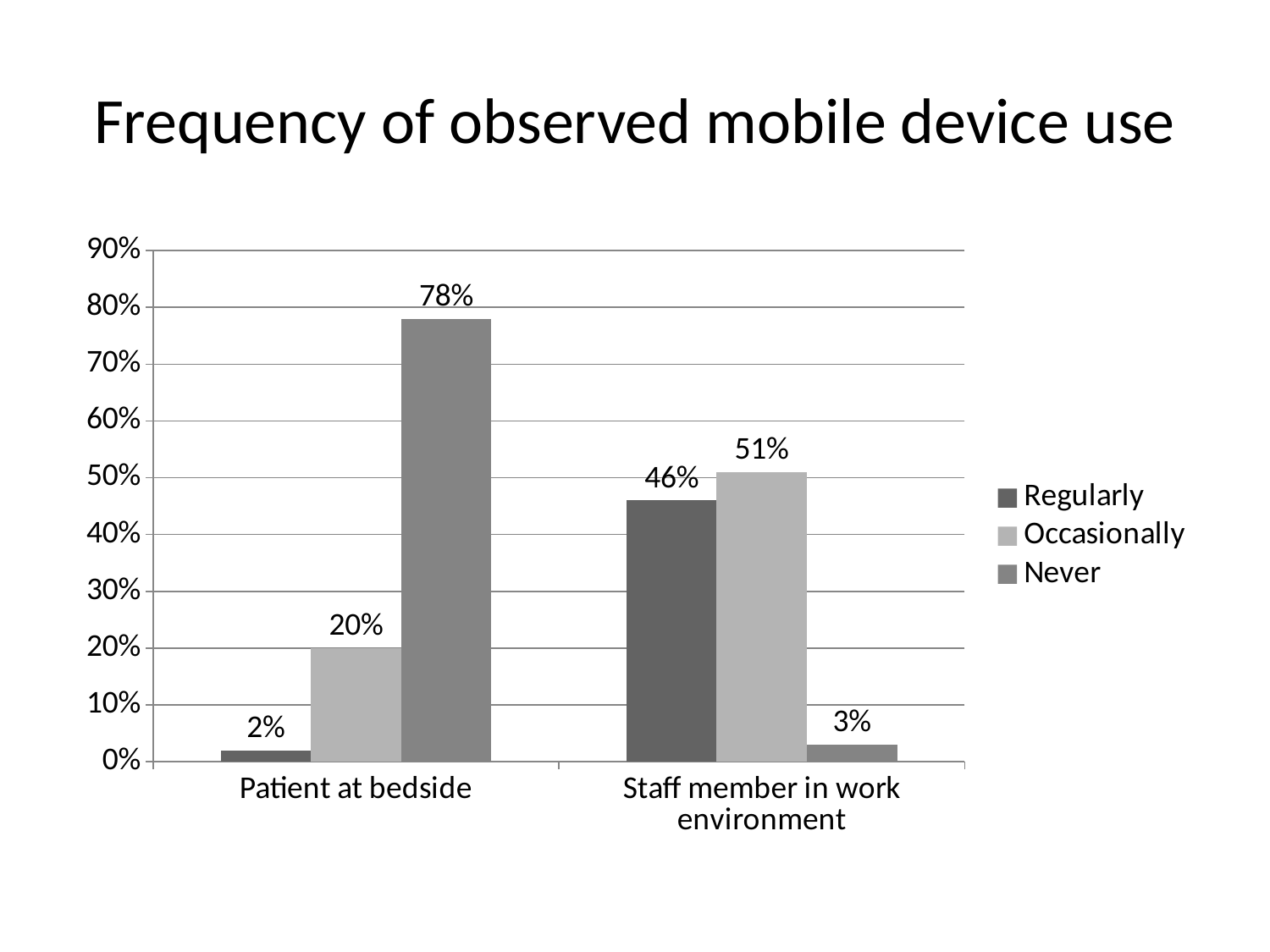

# Frequency of observed mobile device use
### Chart
| Category | Regularly | Occasionally | Never |
|---|---|---|---|
| Patient at bedside | 0.020000000000000004 | 0.2 | 0.78 |
| Staff member in work environment | 0.46 | 0.51 | 0.030000000000000006 |

## Slide 4
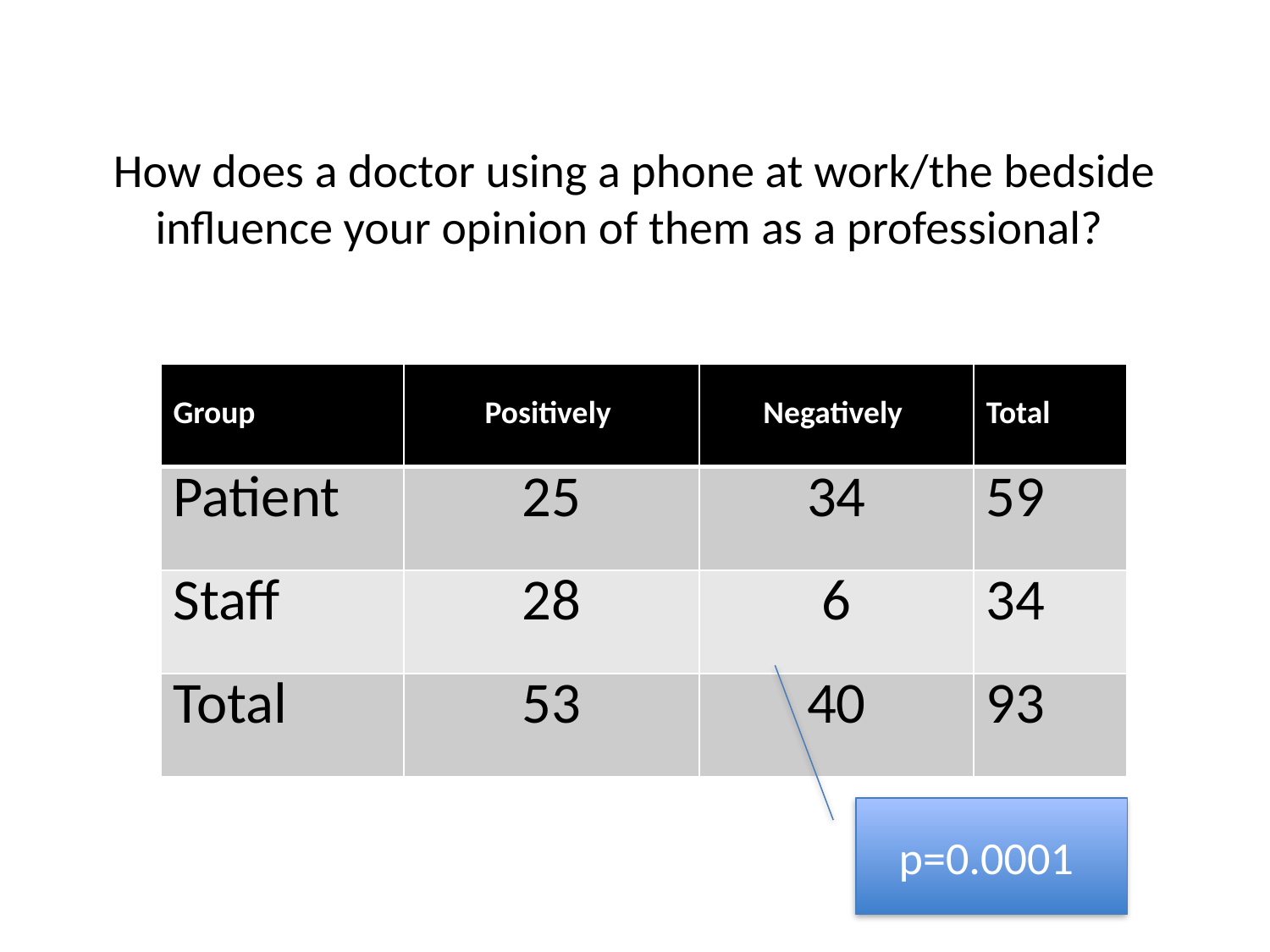

# How does a doctor using a phone at work/the bedside influence your opinion of them as a professional?
| Group | Positively | Negatively | Total |
| --- | --- | --- | --- |
| Patient | 25 | 34 | 59 |
| Staff | 28 | 6 | 34 |
| Total | 53 | 40 | 93 |
p=0.0001
